# Clinical Applications for Diffusion MRI and Tractography of Cranial Nerves Within the Posterior Fossa: A Systematic Review

**DOI:** 10.3389/fnins.2019.00023

**Published:** 2019-02-07

**Authors:** Jonathan Shapey, Sjoerd B. Vos, Tom Vercauteren, Robert Bradford, Shakeel R. Saeed, Sotirios Bisdas, Sebastien Ourselin

**Affiliations:** ^1^Wellcome/EPSRC Centre for Interventional and Surgical Sciences, University College London, London, United Kingdom; ^2^Department of Neurosurgery, National Hospital for Neurology and Neurosurgery, London, United Kingdom; ^3^School of Biomedical Engineering and Imaging Sciences, King's College London, London, United Kingdom; ^4^Translational Imaging Group—Centre for Medical Image Computing, University College London, London, United Kingdom; ^5^Epilepsy Society MRI Unit, Chalfont St Peter, United Kingdom; ^6^The Ear Institute, University College London, London, United Kingdom; ^7^The Royal National Throat, Nose and Ear Hospital, London, United Kingdom; ^8^Neuroimaging Analysis Centre, London, United Kingdom

**Keywords:** MRI—magnetic resonance imaging, diffusion MRI (dMRI), tractography, cranial nerves, brain tumors, trigeminal neuralgia (TN)

## Abstract

**Objective:** This paper presents a systematic review of diffusion MRI (dMRI) and tractography of cranial nerves within the posterior fossa. We assess the effectiveness of the diffusion imaging methods used and examine their clinical applications.

**Methods:** The Pubmed, Web of Science and EMBASE databases were searched from January 1st 1997 to December 11th 2017 to identify relevant publications. Any study reporting the use of diffusion imaging and/or tractography in patients with confirmed cranial nerve pathology was eligible for selection. Study quality was assessed using the Methodological Index for Non-Randomized Studies (MINORS) tool.

**Results:** We included 41 studies comprising 16 studies of patients with trigeminal neuralgia (TN), 22 studies of patients with a posterior fossa tumor and three studies of patients with other pathologies. Most acquisition protocols used single-shot echo planar imaging (88%) with a single b-value of 1,000 s/mm^2^ (78%) but there was significant variation in the number of gradient directions, in-plane resolution, and slice thickness between studies. dMRI of the trigeminal nerve generated interpretable data in all cases. Analysis of diffusivity measurements found significantly lower fractional anisotropy (FA) values within the root entry zone of nerves affected by TN and FA values were significantly lower in patients with multiple sclerosis. Diffusivity values within the trigeminal nerve correlate with the effectiveness of surgical treatment and there is some evidence that pre-operative measurements may be predictive of treatment outcome. Fiber tractography was performed in 30 studies (73%). Most studies evaluating fiber tractography involved patients with a vestibular schwannoma (82%) and focused on generating tractography of the facial nerve to assist with surgical planning. Deterministic tractography using diffusion tensor imaging was performed in 93% of cases but the reported success rate and accuracy of generating fiber tracts from the acquired diffusion data varied considerably.

**Conclusions:** dMRI has the potential to inform our understanding of the microstructural changes that occur within the cranial nerves in various pathologies. Cranial nerve tractography is a promising technique but new avenues of using dMRI should be explored to optimize and improve its reliability.

## Introduction

Diffusion magnetic resonance imaging (dMRI) is a non-invasive magnetic resonance imaging (MRI) technique that is able to provide a quantitative assessment of a tissue's microstructure. dMRI is sensitive to the displacement of water subject to thermally driven Brownian motion and can reveal a tissue's orientational organization (Le Bihan et al., 2001; Basser and Jones, 2002; Jellison et al., 2004). Fiber tractography is a three-dimensional reconstruction of the dMRI data enabling visualization of neural tracts and the brain's connectivity (Mori and van Zijl, 2002; Parker and Alexander, 2005; Gong et al., 2009). Diffusion tensor imaging (DTI) was the initial model to describe the orientational information in dMRI data; it has become a well-established method for imaging the brain's white matter tracts and is now an essential tool in neuroimaging analysis and diagnosis (Assaf and Pasternak, 2008; Ciccarelli et al., 2008). White matter fiber tractography is routinely used in preoperative surgical planning (Nimsky et al., 2005; Yogarajah et al., 2009; Duncan et al., 2016) and may also be incorporated into the neuronavigation system to guide surgery intraoperatively (Coenen et al., 2001; Nimsky et al., 2005; Nowell et al., 2015).

**Figure 1:**
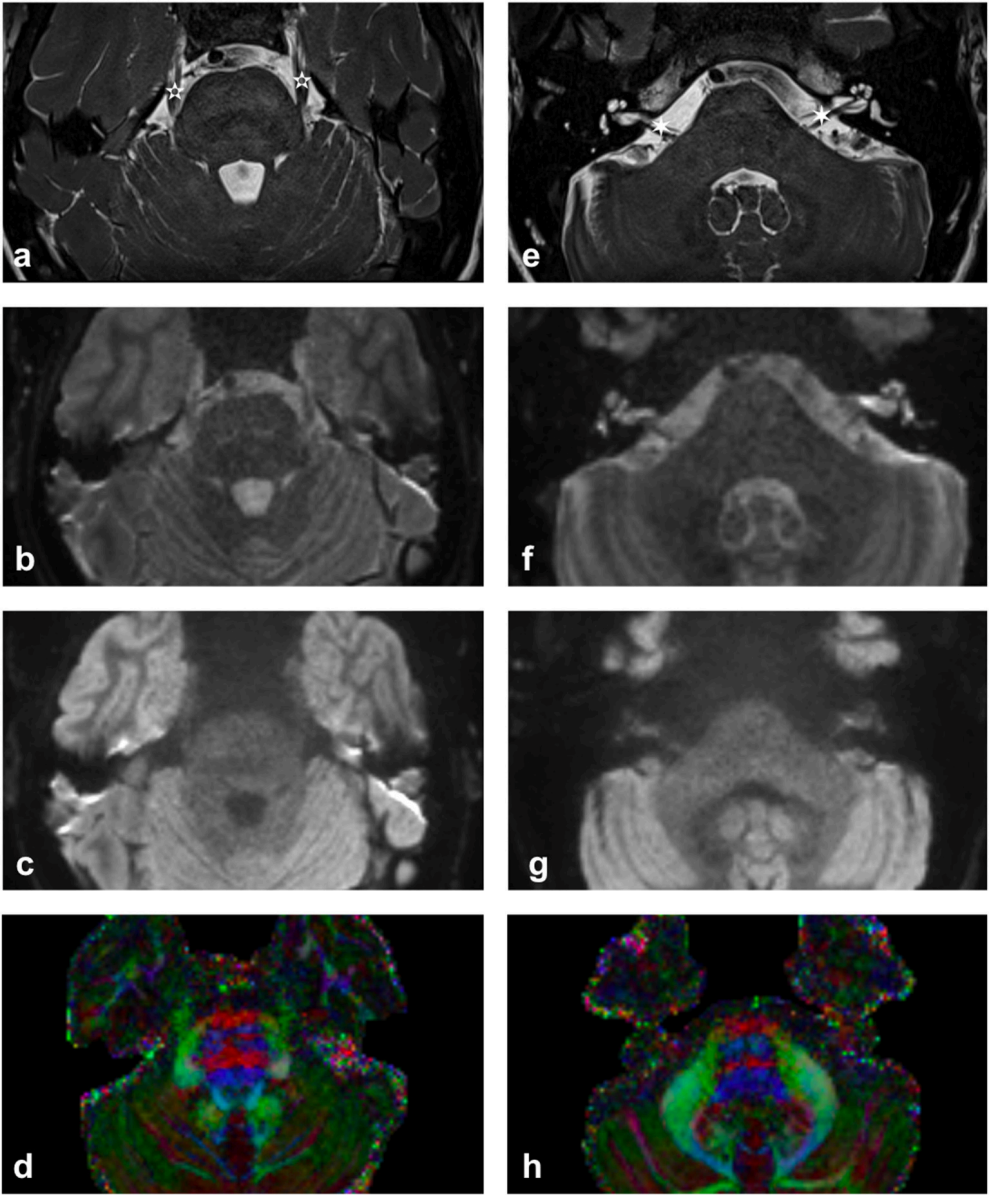
Imaging of upper **(a–d)** and lower **(e–h)** pons. **(a,e)** High contrast T2-weighted images illustrating the trigeminal nerve (white 5-pointed star) and the facial and vestibulocochlear nerves (white 6-pointed star). [acquired with a ZOOMit sequence and a 0.5 × 0.5 × 0.5 mm voxel size]. **(b,f)** mean b0 diffusion weighted image. **(c,g)** mean b1000 diffusion weighted image. **(d,h)** Diffusion-encoded-color map. Note the green anterior trigeminal projections from the brainstem in **(d)** and the red right-left projections of the CN VII/VIII complex in **(h)** [more pronounced on the subject's right-hand side].

More recently, there has been growing clinical interest in utilizing tractography of the cranial nerves in order to assist clinical diagnosis of various neurological pathologies and to inform the surgical planning of neurosurgical procedures such as brain tumor surgery. There are twelve sets of paired cranial nerves (CN I-XII) that typically relay information between the brain and regions of the head and neck and are numbered according to their rostral-caudal position when viewing the brain. The first two cranial nerves—the olfactory [CN I] and optic nerves [CN II] are both sensory nerves, composed of afferent fibers relaying smell and vision, respectively, entering the brain within the anterior and middle cranial fossae. The remaining ten cranial nerves (the oculomotor [CN III], trochlear [CN IV], trigeminal [CN V], abducens [CN VI], facial [CN VII], vestibulocochlear [CN VIII], glossopharyngeal [CN IX], vagus [CN X], spinal accessory [CN XI], and hypoglossal [CN XII]) emerge from the brainstem and course through the posterior fossa and fluid cisterns before exiting the skull (Matsuno et al., 1988). Diffusion MRI and tractography of the cranial nerves in this region is technically challenging due to the nerves' small size (typically 1–5 mm in maximal diameter) and their anatomical location within cerebrospinal fluid (CSF) and close to tissue-air and tissue-bone interfaces (Figure 1).

This paper provides a systematic review of the clinical applications of dMRI and tractography of the cranial nerves within the posterior fossa (CN III-XII). Its aim is to inform clinical readers who are unfamiliar with this imaging modality of the technique's clinical potential, but technical readers with an interest in diffusion imaging form a secondary readership. To aid the reader's understanding, we begin with a brief summary of the biological and physical basis of diffusion MRI and tractography. This is followed by a critical summary and appraisal of different imaging techniques employed that will be of interest to both clinical and technical readers. Finally, we review the effectiveness of these advanced imaging techniques in the context of cranial nerve imaging, examining the various clinical applications for this emerging technology.

## Basic Principles of Diffusion Imaging and Tractography

### Diffusion MRI

Diffusion-weighted MRI creates image contrast based on the relative diffusion of water molecules in tissue. In water, water molecules are able to diffuse freely, and diffusion is equal in all directions (termed *isotropic* diffusion). However, the diffusion of water molecules inside organic tissues is often *anisotropic* (Tanner, 1979) as a result of a tissue's cellular microstructure. In white matter tracts and cranial nerves, diffusion is primarily restricted by axonal membranes, and myelin sheaths causing restricted diffusion perpendicular to the length of the axon with the direction of maximum diffusivity being parallel to the axonal orientation (Moseley et al., 1990).

To sensitize the MRI signal to diffusion, a diffusion-weighting magnetic gradient is applied along a certain axis. Acquiring multiple dMRI images with different diffusion-weighting magnetic gradient orientations can then provide information on the orientation of maximum diffusion. Information concerning the tissue's anisotropy was first described within the *diffusion tensor* framework, which is an abstract mathematical model of diffusion in three-dimensional space (Basser et al., 1994). The tensor model consists of a 3 × 3 matrix derived from diffusivity measurements in at least six non-collinear directions. The diagonal elements (*D*_*xx*_*, D*_*yy*_*, D*_*zz*_) of the tensor represent the diffusion coefficients measured in a frame of reference along each of the principal (x-, y-, and z-) directions. The off-diagonal terms reflect correlation between each pair of principal directions. Conceptually, a diffusion tensor may be visualized as an ellipsoid where the principal major axis is orientated in the direction of maximum diffusivity (Basser et al., 1994). The ellipsoid is characterized by three orthogonal *eigenvectors* (ε_1_, ε_2_, and ε_3_) and its shape determined by three *eigenvalues* (λ_1_, λ_2_, and λ_3_). The eigenvectors represent the major, medium, and minor principal axes of the ellipsoid and the eigenvalues represent the diffusivities in these three directions, respectively (Mori et al., 1999; Basser et al., 2000).

Specific quantitative diffusivity metrics may be calculated from the tensor including axial (AD), radial (RD), and mean diffusivities (MD), as well as a composite metric, fractional anisotropy (FA). The local fiber orientation may also be visualized in a directionally-encoded color (DEC) map of the diffusion tensor that is based on the orientation of the diffusion tensor's first eigenvector (ε_1_). These maps are generated by mapping the major eigenvector's directional components in the x-, y-, and z-planes into RGB color channels and weighting the color brightness by FA.

Fractional anisotropy (FA) is a measure of the coherence of the underlying microstructure and has been shown to correlate both with axonal counts (Schmierer et al., 2007; Gouw et al., 2008) and myelin content (Schmierer et al., 2007). Mean diffusivity (MD) detects the overall diffusion coefficient and is an index proportional to free water and a sensitive marker of inflammation (Werring et al., 1999; Beaulieu, 2002). Another measure of average diffusion is the Apparent Diffusion Coefficient (ADC); the difference being that the ADC is usually derived from the DWI data directly while MD is derived from the tensor fitting on the DWI data. However, as evidenced by the studies included in this review, this terminology is not always strictly followed. Axial diffusivity (AD) indicates diffusion along the main axis of the ellipsoid and radial diffusivity (RD) is a measure of diffusion along the other two orthogonal directions. Animal studies have shown that the AD and RD are good predictors of axonal loss and demyelination, respectively (Song et al., 2002, 2003; Budde et al., 2007) and these have been used as surrogate *in vivo* markers to illustrate axonal integrity (AD) and myelin damage (RD) (Concha et al., 2006; Kraus et al., 2007; Naismith et al., 2009). Despite a high sensitivity to microstructural changes, these quantitative metrics are also affected by factors not incorporated in the diffusion tensor model; such as crossing fibers and other complex fiber architecture, or partial voluming—reducing their specificity (Alexander et al., 2001; Vos et al., 2011, 2012). For a more detailed introduction to DTI and other advanced diffusion methods, readers are referred to a review by Tournier et al. (2011).

### Tractography

The brain's white matter tracts are composed of bundles of axons that share a similar destination and may be delineated using tractography or fiber-tracking algorithms (Parker and Alexander, 2005; Assaf and Pasternak, 2008; Ciccarelli et al., 2008; Gong et al., 2009). Similarly, peripheral nerves are comprised of axons that connect the central nervous system to an end organ. As diffusion imaging and tractography methods have advanced, this technique has been applied to reconstruct ever smaller white matter structures including the cranial nerves.

Tractography uses the voxel-wise information provided by diffusion MRI to infer connections between adjacent voxels that may belong to the same tract to reconstruct the white matter architecture in 3D (Mori and van Zijl, 2002; Lazar et al., 2003). The commonest type of tractography algorithm, *deterministic* tractography, delineates white matter pathways by using an in-line propagation technique whereby data within each voxel directs the tracts subsequent extension. Deterministic tractography is reliant upon three elements: the identification of a suitable starting position to initiate the algorithm (the seed point); continued propagation of the track along the estimated fiber orientation; and the termination of the track when appropriate criteria are met (Mori and van Zijl, 2002; Tournier et al., 2011). Selecting an appropriate seed point is typically performed by the operator but other methods such as selecting the seed point based on functional MR data exist (Tournier et al., 2011). Deterministic DTI tracking uses the first eigenvector of the diffusion tensor to provide a suitable estimate of the fiber orientation within each voxel and then propagates the track according to a fixed user-specified step-size. The most common way of terminating a track is to set a threshold based on a measure of diffusion anisotropy (typically FA) such that if the anisotropy falls below a certain value (e.g., FA < 0.2), the track is not allowed to propagate any further.

*Probabilistic* tractography aims to address the problem of uncertainty of directional information by creating multiple streamlines from a selected distribution of possible fiber orientations with the results presented in the form of a probability distribution, rather than a single “best fit” (Behrens et al., 2007; Tournier et al., 2011). Most probabilistic methods are based on the same underlying model as their deterministic counterparts and so are affected by the same limitations; however, they are able to provide an estimate of the “precision” with which a tract has been reconstructed (Tournier et al., 2011). Several studies examining large matter tracts have demonstrated advantages of using probabilistic tracking over standard deterministic tracking (Farquharson et al., 2013; Li et al., 2013; Lilja et al., 2014; Mandelli et al., 2014) and Rueckriegel et al. recently described the benefits of probabilistic tracking to depict the auditory pathway in cases of vestibular schwannoma (Rueckriegel et al., 2016).

## Methods

The Preferred Reporting Items for Systematic Reviews and Meta-Analyses (PRISMA) Statement was used in the preparation of this manuscript (Moher et al., 2009) and the study was registered with PROSPERO: an international prospective register of systematic reviews (CRD117068).

A structured search of the Pubmed, Web of Science and EMBASE databases was undertaken over a 20-year period. The last date of the search was December 11th, 2017. Two independent researchers applied the search criteria using the Boolean search terms:

1 (diffusion tensor imaging OR diffusion MRI OR diffusion tensor tracking OR tractography OR fiber tracking OR fiber tracking)

AND

2 (oculomotor nerve OR trochlear nerve OR trigeminal nerve OR abducens nerve OR facial nerve OR vestibular nerve OR vestibulocochlear nerve OR cochlear nerve OR vestibulocochlear complex OR facial-vestibulocochlear complex OR glossopharyngeal nerve OR vagus nerve OR accessory nerve OR hypoglossal nerve OR cranial nerve)

Reference lists of included articles were also reviewed, and expert opinion sought, to identify further eligible publications.

Eligibility for inclusion in the systematic review included peer-reviewed publications in which English-language manuscripts were available through electronic indexing comprising:

1. Clinical studies of patients with associated cranial nerve pathology.
2. Diffusion MRI and/or fiber tractography of lower cranial nerve(s) has been performed.
3. The diffusion imaging technique used has been described.
4. The imaging success rate is reported.

Full articles were obtained and further assessed for eligibility and any discrepancy was resolved through mutual review and involvement of the senior author.

**Figure 2:**
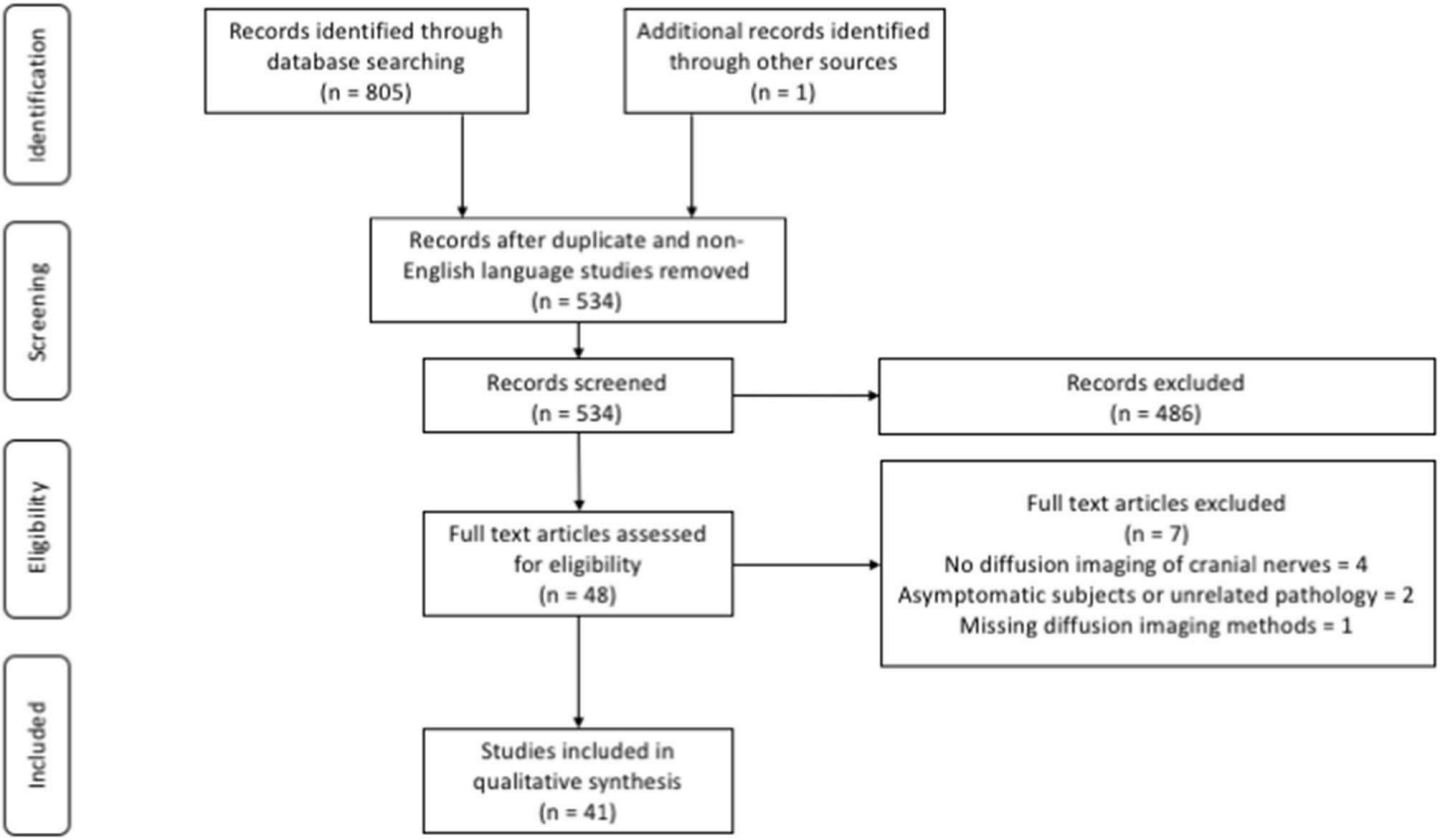
PRISMA flow diagram of article selection.

In total, 805 articles were identified through the database searches and an additional record was identified through other sources. Following removal of duplicate and non-English language studies, 534 manuscript titles and abstracts were screened. After applying the eligibility criteria 48 full text articles were reviewed and a further 7 articles were excluded. In all, 41 studies satisfied the inclusion criteria and were included in further qualitative analysis (Figure 2).

Data extraction was performed using a table with a predefined set of criteria including:

1. Study design.
2. Study group characteristics, including the number of patients, duration of symptoms (in cases of trigeminal neuralgia), tumor characteristics (where applicable), and treatment modality.
3. Imaging acquisition details, including hardware, data specifics (including sequence(s), number of directions (number of signal averages), acquired voxel size, b-value(s), scan time), and software.
4. Diffusion processing methods, including region of interest, fiber tractography method (type [deterministic or probabilistic], number of ROI(s), Fractional anisotropy threshold.
5. Effectiveness of dMRI analysis, including success rate in generating tractography results and diffusivity measurements of the target cranial nerve(s). In surgical cases, the method and results of any intraoperative validation was also noted.
6. Key findings.

The methodological quality of the included studies was assessed by using the Methodological Index for Non-Randomized Studies (MINORS) scoring system for observational studies (Slim et al., 2003). Observational studies include comparative studies such as case-control and cohort designs, and patient series which may or may not involve comparisons between groups. All studies are scored on the following criteria: (1) A stated aim of the study; (2) Inclusion of consecutive patients; (3) Prospective collection of data; (4) Endpoint appropriate to the study aim; (5) Unbiased evaluation of endpoints; (6) Follow-up period appropriate to the major endpoint; (7) Loss to follow-up not exceeding 5%; (8) Prospective calculation of the study sample size. Comparative studies are also scored with respect to; (9) An adequate control group; (10) Contemporary groups; (11) Baseline equivalence of groups; (12) Adequate statistical analyses. Rating scores out of 16 and 24 for non-comparative and comparative studies, respectively, were generated by the lead author. Studies of greater quality, i.e., those with a higher MINORS score, were given greater weighting in the subsequent qualitative synthesis.

## Results

Forty-one studies satisfied the inclusion criteria and underwent qualitative analysis, including 20 case series, 15 non-randomized case-control studies, 4 cohort studies and 2 case reports. A maximum of 959 participants were included across all studies (ranging from 1 to 150 subjects per study), if multiple studies form a single institution did not include overlapping patient groups. Twenty-two studies focused on imaging the cranial nerves in relation to a posterior fossa tumor (Taoka et al., 2006; Chen et al., 2011; Gerganov et al., 2011; Roundy et al., 2012; Zhang et al., 2013, 2017; Choi et al., 2014; Ulrich et al., 2014; Wei et al., 2015, 2016; Yoshino et al., 2015a,b, 2016; Borkar et al., 2016; Hilly et al., 2016; Ma et al., 2016; Song et al., 2016; Behan et al., 2017; d'Almeida et al., 2017; Li et al., 2017; Zolal et al., 2017a,b), 16 studies imaged the trigeminal nerve in patients with trigeminal neuralgia (Herweh et al., 2007; Fujiwara et al., 2011; Leal et al., 2011; Lutz et al., 2011, 2016; Hodaie et al., 2012; Liu et al., 2013; Wilcox et al., 2013; DeSouza et al., 2014, 2015; Lummel et al., 2015; Chen, D. Q. et al., 2016; Chen, S. T. et al., 2016; Lin et al., 2016; Neetu et al., 2016; Hung et al., 2017) and the remaining 3 studies evaluated DTI and tractography of the cranial nerves in various other pathologies including the cochlear nerve in cases of unilateral deafness (Vos et al., 2015), and the trigeminal nerve in patients with herpetic keratouveitis (Rousseau et al., 2015) and short lasting unilateral neuralgiform headache attacks with conjunctival injection and tearing (SUNCT) (Coskun et al., 2017) (Tables 1–3).

**Table 1:** Diffusion imaging and tractography of the trigeminal nerve in trigeminal neuralgia.

**References**	**Study design**	**MINORS**	**Participants**				**Hardware**	**Diffusion MRI data**					**Software**	**ROI**	**Fiber tractography method**			**Effectiveness & results**		
			***n***	**Cohorts**	**Median symptom duration (patients)**	**Treatment modality**	**Scanner**	**Seq**.	**Dir. (NSA)**	**Acq'd voxel size (mm)**	**b-value (s/mm^**2**^)**	**Scan time**	**Software (Model)**		**Type**	**ROI**	**FA-t**	**Tract-ography success rate**	**dMRI success rate**	**Key findings**
Herweh et al., 2007	Case-control	14/24	13	NVC-TN *(n* = 6)	8y (2–15y)	MVD	1.5T Symphony, Siemens	Flair-EPI	6 (1)	2.5 × 2.5 × 3.0	1,000	2′41″	MatLab (DTI)	REZ M NS	N/A	N/A	N/A	N/A	13/13 (100%)	FA lower in TN-affected nerves vs. unaffected side in 3/6 (50%) of patients
				HC *(n* = 7)	N/A	N/A													
Fujiwara et al., 2011	Case-control	22/24	27	NVC-TN *(n* = 13) HC *(n* = 14)	2y (3m−7y) N/A	MVD & GKRS *(n* = NS) N/A	3T Signa, GE	Single-shot EPI	6 (8)	1.6 × 1.6 × 1.2	1,000	8′30″	dTV (DTI)	CIS M Diff	D	1	0.1	27/27 (100%)	27/27 (100%)	No significant difference in FA or ADC values when TN-affected nerves compared with patients' unaffected side or vs. HC. Positive correlation & reduction in FA following MVD (*p* < 0.01) [*n* = 3]
Leal et al., 2011	Case-control	22/24	16	NVC-TN *(n* = 10) HC *(n* = 6)	5y (3.5–13y) N/A	MVD N/A	3T Achieva, Philips	Single-shot EPI	32 (1)	2.0 × 2.0 × 2.0	1,000	6′02″	MedINRIA (DTI)	REZ M Diff	N/A	N/A	N/A	N/A	16/16 (100%)	FA significantly lower in TN-affected nerves vs. unaffected side & vs. HC (*p* < 0.05). ADC significantly higher in TN-affected nerves vs. affected side & vs. control group (*p* < 0.05)
Lutz et al., 2011	Case-control	22/24	20	NVC-TN *(n* = 20)	4y (1–10y)	MVD	3T Signa, GE	Single-shot EPI	15 (10)	1.6 × 1.6 × 2.0	1,000	11′33	FuncTool (DTI)	REZ M An	N/A	N/A	N/A	N/A	20/20 (100%)	FA significantly lower in TN-affected nerves vs. unaffected side (*p* = 0.004). ADC values nearly identical in both groups
Hodaie et al., 2012	Case series	16/24	5	NVC-TN *(n* = 5)	NS	GKRS	3T Signa, GE	Single-shot EPI	25(1)	NS (ST: 3.0)	1,000	NS	3D Slicer (DTI)	CIS* M NS	D	2	0.2	5/5 (100%)	5/5 (100%)	Treatment with GKRS resulted in significant changes in FA & RD at the “target” ROI [A 47% decrease in FA values (*p* = 0.027) & a 55.8% increase in RD values (*p* = 0.002). No change in AD]
Liu et al., 2013	Case-control	18/24	22	NVC-TN *(n* = 16) HC *(n* = 6)	5y (2–12y) N/A	NS N/A	3T Trio, Siemens	Single-shot Flair-EPI	12 (1)	1.9 × 1.9 × 3.0	1,000	11′46″	Leonardo syngo 2003A (DTI)	REZ M Diff	N/A	N/A	N/A	N/A	22/22 (100%)	FA significantly lower in TN-affected nerves vs. unaffected side & vs. HC. No significant changes in AD. Trend toward significantly higher MD in TN-affected nerves vs. unaffected side
Wilcox et al., 2013	Case-control	16/24	73	NVC-TN *(n* = 9) Neur-opathy *(n* = 18) TMD *(n* = 20) HC *(n* = 26)	7y (2–35y) 3.5y (1.3–11y) 5.25y (1.5–30y) N/A	Meds only N/A	3T Achieva, Philips	Single-shot EPI	32 (4)	2.0 × 2.0 × 2.5	1,000	NS	SPM8 (DTI)	REZ M An	N/A	N/A	N/A	N/A	73/73 (100%)	No significant difference in FA or MD values.in TN-affected nerves vs. unaffected side. No change in DTI values observed in other pathologies.
DeSouza et al., 2014	Case-control	17/24	36	NVC-TN *(n* = 18) HC *(n* = 18)	NS N/A	NS N/A	3T Philips (Model NS)	Single-shot EPI	60 (1)	1.9 × 1.9 × 3.0	1,000	NS	FSL (DTI)	REZ M An	D	1	0.2	36/36 (100%)	36/36 (100%)	FA significantly lower in TN-affected nerves vs. unaffected side & vs. HC (*p* < 0.05). No significant difference in MD, RD or AD in TN-affected nerves vs. unaffected side. Higher MD, RD & AD found bilaterally in TN-patients vs. HC (*p* < 0.05)
DeSouza et al., 2015	Case-control	17/24	28	NVC-TN *(n* = 14) HC *(n* = 14)	5.5y (1–30y) N/A	MVD *(n* = 7) GKRS *(n* = 7) N/A	3T Signa, GE	Single-shot EPI	60 (1)	0.94 × 0.94 × 3.0	1,000	NS	FSL (DTI)	REZ M An	N/A	N/A	N/A	N/A	14/14 (100%)	FA significantly lower & MD, RD & AD significantly higher in TN-affected nerves vs. HC (*p* < 0.05). Effective treatment reversed FA, MD, RD & AD abnormalities & correlated with pain relief after treatment
Lummel et al., 2015	Case-control	22/24	36	MS-TN *(n* = 12) NVC-TN *(n* = 12) HC *(n* = 12)	7 (0.5–11y) 5y (8m−11y) N/A	NS NS N/A	3T Signa, GE	Single-shot EPI	15 (10)	1.7 × 1.7 × 2.0	1,000	11′55″	FuncTool (DTI)	REZ M An	N/A	N/A	N/A	N/A	36/36 (100%)	In NVC-TN, FA significantly lower in TN-affected nerves vs. unaffected side (*p* = 0.002) & vs. HC (*p* < 0.001). ADC significantly higher on TN-affected side vs. unaffected side (*p* = 0.007) & vs. HC (*p* = 0.005). In MS-TN, DTI reveals microstructural changes on both the TN-affected and unaffected sides.
Chen, D. Q. et al., 2016	Case-control	16/24	30	MS-TN *(n* = 10) NVC-TN *(n* = 10) HC *(n* = 10)	NS	NS NS N/A	3T Signa, GE	Single-shot EPI	60 (1)	1.9 × 1.9 × 3.0	1,000	NS	3D Slicer (DTI)	CIS REZ Pons M An	D	1	0.2	SDT:“unsuccessful” MTT: “successful”	30/30 (100%)	Underlying TN-pathology alters the diffusivity pattern of different nerve segments.In NVC-TN, TN-affected nerves showed higher FA in the cisternal segment of & lower FA in the REZ segment vs. unaffected side & vs. controls (*p* < 0.05).In MS-TN, FA was lower in the peri-lesional segments of TN-affected nerves. No significant differences in MD, RD & AD noted.
Chen, S. T. et al., 2016	Case series	19/24	43	NVC-TN *(n* = 43)	5y	RFA	3T Verio, Siemens	Multi-shot EPI (5)	30 (1)	2.0 × 2.0 × 2.0	1,000	20′58″	DSI Studio (DTI)	CIS M An	N/A	N/A	N/A	N/A	43/43 (100%)	FA significantly lower (*p* < 0.001), ADC higher (*p* = 0.006) & RD lower (*p* < 0.001) in TN-affected nerves vs. unaffected side. No difference in AD. Trend toward FA reduction in effective treatment responders (*p* = 0.072)
Lin et al., 2016	Case-control	15/24	150	NVC-TN *(n* = 50) N-NVC-TN (n=50) HC (n=50)	NS	N/A	3T Signa, GE	Single-shot EPI	20 (1)	1.9 × 1.9 × 2.4	1000	5′12″	FuncTool (DTI)	REZMNS	N/A	N/A	N/A	N/A	150/150 (100%)	RD significantly higher in TN-affected nerves vs. unaffected side (*p* = 0.00) & HC (*p* = 0.00). No difference in AD.
Lutz et al., 2016	Case series	20/24	81	NVC-TN *(n* = 81)	13y (5m−21y)	MVD	3T signa, GE	Single-shot EPI	NS (4)	1.7 × 1.7 × 2.0	NS	11′55″	FuncTool (DTI)	REZ M An	N/A	N/A	N/A	N/A	81/81 (100%)	FA significantly lower in TN-affected nerves vs. unaffected side (*p* = 0.005). No significant difference in ADC values (*p* = 0.092)
Neetu et al., 2016	Case-control	17/24	8	N-NVC-TN *(n* = 4) HC *(n* = 4)	5w N/A	None N/A	3T Signa, GE	Single-shot EPI	15 (1)	1.09 × 1.09 × 1.6	1000	NS	FuncTool (DTI)	REZ M An	N/A	N/A	N/A	N/A	8/8 (100%)	FA significantly lower (*p* = 0.001) & ADC higher (*p* = 0.001) in TN-affected nerves vs. unaffected side. & vs. HC.
Hung et al., 2017	Case-control	19/24	47	NVC-TN-R *(n* = 17) NVC-TN-NR *(n =* 14) HC *(n* = 16)	NS NS N/A	MVD *(n =* 10) GKRS *(n =* 21) N/A	3T Signa, GE	Single-shot EPI	60 (1)	1.9 × 1.9 × 3.0	1000	NS	3D Slicer (DTI)	CIS REZ Pons M NS	D	1	0.2	47/47 (100%)	47/47 (100%)	Treatment outcomes may be predicted by pre-surgical diffusivity alterations: Long-term responders have lower cisternal segment AD & MD values; Non-responders have abnormalities located more centrally with lower FA values in the REZ & higher AD values in the pontine segment

*Seq., Sequence; Dir., Directions; NSA, Number of signal average; ROI, Region of Interest; NVC-TN, Neurovascular compression associated trigeminal neuralgia; MS-TN, Multiple Sclerosis associated trigeminal neuralgia; HC, Healthy control; N-NVC-TN, Non-neurovascular compression associated trigeminal neuralgia; N/A, Not applicable; NS, Not specified; MVD, Microvascular Decompression; GKRS, Gamma Knife Radiosurgery; EPI, Echo planar imaging; DTI, Diffusion Tensor Imaging; REZ, Root Entry Zone; CIS; Cisternal segment; M, manual placement of ROI; A, automated placement of ROI; An, ROI placed using anatomical image fused with diffusion data; Diff, ROI placed using diffusion data only; D, Deterministic; SDT, Single-tensor diffusion tractography; MTT, Multi-tensor tractography; FA-t: Fractional anisotropy threshold; FA, Fractional anisotropy; ADC, Apparent Diffusion Coefficient; MD, Mean diffusivity; RD, Radial diffusivity; AD, Axial diffusivity*.

*Two distinct ROIs within the cisternal segment were analyzed including the radiosurgical target region and an ROI proximal to the root entry zone (but still within the cisternal segment of the nerve).

**Table 2:** Diffusion imaging and tractography of cranial nerves in other pathologies.

**References**	**Study design**	**MINORS**	**Patients**		**Hardware**	**Diffusion MRI data**					**Software**	**Fiber tractography method**				**Effectiveness & results**		
			***n***	**Cohorts**	**Scanner**	**Seq**.	**Dir. (NSA)**	**Acq'd voxel size (mm)**	**b-value (s/mm^**2**^)**	**Scan time**	**Software (model)**	**Type**	**ROI**	**FA-t**	**CNs**	**Tractography success rate**	**dMIR success rate**	**Key findings**
Rousseau et al., 2015	Case-control	22/24	36	Herpetic Keratouveitis*(n* = 12)	1.5T Sonata, Siemens	Single-shot EPI	25 (3)	1.9 × 1.9 × 2.0	500	7′30″	MedINRIA (DTI)	D	CIS M Diff	0.2	V	36/36 (100%)	36/36 (100%)	FA significantly lower in REZ of trigeminal nerves on the ipsilateral side to the affected eye vs. unaffected side (*p* = 0.03)
				HC *(n* = 24)													
Vos et al., 2015	Case-control	20/24	10	Unilateral deafness *(n* = 5)	3T Achieva, Philips	Single-shot EPI	22 (2)	1.8 × 1.8 × 1.8	1,000	7′49″	ExploreDTI (DTI)	D	Coch P-sag plane M An	NS	VIII	10/10 (100%)	10/10 (100%)	Significantly lower FA in Cochlea n. of both deaf side (*p* = 0.030) & healthy side (*p* = 0.013) in patients vs. HC. No statistical difference in FA or MD between deaf and healthy sides in patient group
				HC *(n* = 5)													
Coskun et al., 2017	Case series	5/16	2	SUNCT Headache *(n* = 2)	3T Verio, Siemens	SIngle-shot EPI	30 (1)	1.8 × 1.8 × 4.0	1,000	NS	NEURO 3D (DTI)	D	CIS M Diff	NS	V	2/2 (100%)	2/2 (100%)	FA lower on affected side. ADC value higher on affected side 1 patient but no difference was observed in the other

*Seq., Sequence; Dir., Directions; NSA, Number of signal average; Diff., Diffusion; ROI, Region of Interest; HC, Healthy control; SUNCT, Short-lasting unilateral neuralgiform headache with conjunctival injection and tearing (SUNCT syndrome); EPI, Echo planar imaging; D, Deterministic; DTI, Diffusion Tensor Imaging; REZ, Root Entry Zone; M, manual placement of ROI; A, automated placement of ROI; An, ROI placed using anatomical image fused with diffusion data; Diff, ROI placed using diffusion data only; FA-t, Fractional anisotropy threshold; CIS, Cisternal segment; Coch, Cochlea; P-sag plane, 10 voxels (18 mm) to left and right of mid-saggital plane; FA, Fractional anisotropy; ADC, Apparent Diffusion Coefficient; MD, Mean diffusivity*.

**Table 3:** Diffusion imaging and tractography of cranial nerves in patients with posterior fossa tumors.

**References**	**Study design**	**MINORS**	**Patients**				**Hardware**	**Diffusion MRI data**					**Software**	**Fiber tractography method**			**CNs**	**Effectiveness & results**			
			***n***	**Path**	**Median tumor size**	**Median volume (mm^**3**^)**	**Scanner**	**Seq**.	**Dir. (NSA)**	**Acq'd voxel size (mm)**	**b-value (s/mm^**2**^)**	**Scan time**	**Software (model)**	**Type**	**ROI**	**FA-t**	**CNs**	**Tractography success rate**		**Intra-operative correlation method**	**Intra-operative accuracy rate**	**Prevention of facial nerve injury**
Taoka et al., 2006	Case series	11/16	8	VS	25.5 mm (18–47 mm)	NS	1.5T Sonata, Siemens	Single-shot EPI	6 (6)	1.8 × 1.8 × 3.0	1,000	NS	dTV-II (DTI)	D	IAM M Diff	0.10	VII	7/8 (88%)		Inspection by surgeon & qualitative EP monitoring	5/7 (71%)	Not assessed
Chen et al., 2011	Case series	4/16	3	VS	NS (10–30 mm)	8,036.72	3T SIGNA HDx, GE	Single-shot EPI	25 (1)	NS (ST: 3.0)	1,000	NS	3D Slicer (DTI)	D	REZ/ IC M NS	0.2	V, VII-VIII	3/3 (100%)		Not assessed	Not assessed	Not assessed
Gerganov et al., 2011	Case series	14/16	22	VS	26.0 mm (16–50 mm)	NS	3T Allegra, Siemens	Single-shot EPI	12 (1)	NS ST: 1.6	1,000	NS	iPLAN (DTI)	D	IAM REZ M An	0.10	VII	22/22 (100%)		Inspection by surgeon & qualitative EP monitoring	20/22 (91%)	Not assessed. Facial nerve function 2 weeks after surgery: HB I-II: 14/22 (64%), HB III+: 8/22 (36%)
Roundy et al., 2012	Cohort	17/24	5	VS *(n = 2)* NS *(n = 3)*	35.0 mm (25–52 mm)	NS	3T Philips	Single-shot EPI	S-DTI: 6 (1)	1.8 × 1.8 × 4	1,000	42″	Track Vis (DTI) Philips Software (DTI)	D	Tum M An	0.15	VII	S-DTI: 0/5 (0%)		Inspection by surgeon & qualitative EP monitoring	S-DTI: N/A	Not assessed
									HD-DTI: 32 (2)	1.6 × 1.6 × 1.2	1,000	9′39″						HD DTI 5/5 (100%)			HD DTI: 5/5 (100%)	
Zhang et al., 2013	Case series	12/16	8	VS	38.1 mm (30-55mm)	NS	3T Verio, Siemens	Single-shot EPI	NS (1)	1.8 × 1.8 × 3.0	1,000	NS	NS	D	IAM BS M NS	0.10	VII	7/8 (87.5%)		Inspection by surgeon & qualitative EP monitoring	7/7 (100%)	Not assessed. Facial nerve function 9-12 months after surgery: HB I-II: 8/8 (100%)
Choi et al., 2014	Case series	10/16	11	VS	22.0 mm (18–38 mm)	NS	1.5T Gyroscan Intera, Philips	Single-shot EPI	32 (1)	2.5 × 2.5 × 2.5	1,000	NS	DTI-studio (DTI)	D	IAM REZ M NS	V	VII	11/11 (100%)		Inspection by surgeon & qualitative EP monitoring	11/11 (100%)	Not assessed. Facial nerve function 12 months after surgery: HB I-II: 9/11 (82%), HB III+: 2/11 (18%)
Ulrich et al., 2014	Case series	6/16	7	BC	NS		3T Achieva, Philips	Single-shot EPI	15 (2)	2.1 × 2.1 × 2.1	1,000	5′00″	Philips Software (DTI)	D	NS	NS	V,-VII	7/7 (100%)		Not assessed	Not assessed	Not assessed
Wei et al., 2015	Case series	12/16	23	VS	34.0 mm (16–50 mm)	NS	3T Trio, Siemems	Single-shot EPI	30 (1)	NS (ST: 2.0)	NS	NS	iPlan (DTI)	D	IAM MC M An	V	V-VII	23/23(100%)		Inspection by surgeon & qualitative EP monitoring	21/23 (91%)	Not assessed. Facial nerve function 6 months after surgery: HB I-II: 16/23 (70%), HB III+: 7/23 (30%)
Yoshino et al., 2015a	Case series	7/16	11	VS	28.8 mm (20–40 mm)	NS	3T Signa, GE	Single-shot EPI	30 (1)	2.0 × 2.0 × 2.5	1,000	NS	dTV-II SR (DTI)	D	IAM M Diff	V	VII, VIII	10/11 (82%)		Inspection by surgeon & qualitative EP monitoring	3/10 (30%)	Not assessed. Facial nerve function after surgery (no date specified): HB I-II: 11/11 (100%)
Yoshino et al., 2015b	Cohort	16/24	22	VS	28.7 mm (9–52 mm)	NS	3T Signa, GE	Single-shot EPI	30 (1)	2.0 × 2.0 × 2.5	1,000	NS	dTV-II SR (DTI)	D	IAM M NS	V	VII, VIII	Standard DTI: 18/22 (82%) DTT-CE-FIESTA: 21/22 (95%)		Inspection by surgeon & qualitative EP monitoring	Standard DTI: 3/18 (17%) DTT-CE-FIESTA: 14/21 (67%)	Not assessed. Facial nerve function after surgery (no date specified): HB I-II: 21/22 (95%), HB III+: 1/22 (5%)
Borkar et al., 2016	Case series	8/16	20	VS	44.0 mm (30–61 mm)*	NS	3T Achieva, Philips	Single-shot EPI	15 (3)	0.78 × 0.78 × 1.5	800	NS	Dyna-Suite Neuro 3.0 (DTI)	D	IAM REZ M An	0.12	VII	19/20 (95%)		Inspection by surgeon	16/18 (89%) [the facial nerve was not identified intraoperatively in 1 patient]	Not assessed
Hilly et al., 2016	Case series	7/16	21	VS	44.0 mm (30–61mm)	NS	3T Achieva, Philips	Single-shotEPI	32 (1)	2.0 × 2.0 × 2.0	NS	NS	FiberTrak (DTI)	D	NS	0.10	VII	20/21 (95%)		Inspection by surgeon	Initial results: 15/20 (75%) Discordant results “re-evaluated” and revised agreement reported: 19/20 (95%)	Not assessed
Wei et al., 2016	Case series	7/16	3	TS	43.0 mm (18–61 mm)	NS	3T Trio, Siemens	Single-shot EPI	30 (1)	NS (ST: 2.0)	NS	NS	iPLAN (DTI)	D	IAM/MC BS M An	NS	V-VIII	12/12 CNs (100%)		Inspection by surgeon & qualitative EP monitoring (CN VII & VIII)	11/12 CNs (92%)	Not assessed. Normal facial function & improved hearing documented in 1 patient at 12 months FU.
Yoshino et al., 2016	Case report	10/16	1	PM	30.0 mm		3T Trio, Siemens	Single-shot EPI	101 (1)	2.4 × 2.4 × 2.4	x12 b-values (max 5,000)	15′00″	DSI-studio (DSI)	D	NS	V	III-VI	CN III-VI (100%)		Inspection by surgeon	1/1 (100%)	Not assessed
Ma et al., 2016	Case series	11/16	12	VS *(n* = 9) M *(n* = 3)	40.0 mm (31–46mm)	NS	3T GE	Single-shot EPI	32 (1)	1.6 × 1.6 × 2.0	1,000	NS	3D Slicer (DTI)	D	NS M An	0.18	IV-VIII	11/12 (92%)		Inspection by surgeon & qualitative EP monitoring	11/11 (100%)	Not assessed
Song et al., 2016	Case series	10/16	15	VS	35.0 mm (15–45 mm)	NS	3T Discovery 750, GE	Single-shot EPI	30 (2)	1.6 × 1.6 × 1.2	1,000	9′00″	3D Slicer (DTI)	D	IAM REZ M An	0.10	VII	14/15 (93%)		Inspection by surgeon & qualitative EP monitoring	13/14 (93%)	Not assessed
d'Almeida et al., 2017	Case report	4/16	1	PM	NS		1.5T Avanto, Siemens	Single-shot EPI	6 (4)	1.8 × 1.8 × 5.0	1,000	NS	dTV-II (DTI)	D	NS	NS	VII	1/1 (100%)		Inspection by surgeon & qualitative EP monitoring	1/1 (100%)	Not assessed
Zhang et al., 2017	Case series	14/16	30	VS	33.3 mm (20–54 mm)	NS	3T Discovery 750, GE	Single-shot EPI	64 (1)	NS (ST: 2.0)	1,000	NS	iPlan (DTI)	D	IAM REZ M An	V	VII	30/30 (100%)		Inspection by surgeon & qualitative EP monitoring	29/30 (97%)	Not assessed. Facial nerve function 2 weeks after surgery: HB I-II: 21/30 (70%), HB III+: 9/30 (30%)
Zolal et al., 2017a	Cohort	16/24	3	VS *(n* = 2) M *(n* = 1)	NS	NS	3T Verio, Siemens	Multi-shot EPI (NS)	20 (1)	2.0 × 2.0 × 2.0	800	12′00″	D: DSI-Studio (DSI) P:FSL (DTI)	D P	RG/ IAMBS M An	V V	V, VII, VIII	3/3 (100%) 3/3 (100%)		Inspection by surgeon	1/3 (30%) 3/3 (100%)	Not assessed
Zolal et al., 2017b	Case series	11/16	21	VS	NS	3,326.8	3T Verio, Siemens	Multi-shot EPI (NS)	20 (1)	NS (ST: 2.0)	800	NS	FSL (DTI)	P	IAM BS-C M An	V	VII, VIII	21/21 (100%) One pathway: 7/21 Two pathways: 14/21		Inspection by surgeon & qualitative EP monitoring	17/21 (81%)	Not assessed. Facial nerve function 8 days after surgery: HB I-II: 16/21 (76%%), HB III+: 5/21 (24%)
Li et al., 2017	Case series	14/16	19	VS	39.8 mm (25–53 mm)	NS	3T Trio, Siemens	Single-shot EPI	32 (1)	1.8 × 1.8 × 1.0	1,000	NS	StealthViz (DTI)	D	IAM REZ M NS	V	VII	18/19 (95%)		Inspection by surgeon & quantitative EP stimulation recorded with integrated neuro-navigation	17/18 (94%)	Not assessed. Facial nerve function 12 months after surgery: HB I-II: 18/19 (95%), HB III+: 1/19 (5%)
Behan et al., 2017	Cohort	14/24	10	VS *(n* = 6) M *(n* = 3) TS *(n* = 1)	19.5 mm (11.5–29.0 mm)	1,285.8	3T Signa HDx, GE	Single-shot EPI	60 (1)	1.9 × 1.9 × 3.0	1,000	17′30″	3D Slicer (DTI) XST: TEEM (DTI) MRtrix3 (CSD)	D	RG IC M An	0.15	V, VII, VIII	CN V 4/4 (100%)4/4 (100%) 4/4 (100%)	CN VII/VIII 5/6 (83%) 6/6 (100%) 6/6 (100%)	Not assessed	Not assessed	Not assessed

*Path., Pathology; Seq., Sequence; Dir., Directions; NSA, Number of signal average; Diff., Diffusion; ROI, Region of Interest VS, Vestibular schwannoma; NS, Not specified; ST, Slice thickness; EPI, Echo planar imaging; NSA, Number of signal average; DTI, Diffusion Tensor Imaging; DSI, Diffusion spectrum imaging; D, Deterministic; P, Probabilistic CSD, Constrained spherical deconvolution; ROI, Region of Interest; M, manual placement of ROI; A, automated placement of ROI; An, ROI placed using anatomical image fused with diffusion data; Diff, ROI placed using diffusion data only; IAM, Internal Auditory Meatus; Tum, ROI drawn through mid-saggital plane through tumor in a midcisternal location; MC, Meckel's cave; REZ, Root entry zone; RG, retrogasserian ganglion; IC, intracanalicular ROI; BS, Brainstem surface; BS-C, Brainstem surface corresponding to position of facial/cochlear nerve of the other side; FA-t, Fractional anisotropy threshold; V, Variable; HD, High definition; EP, Electrophysiolgogy; HB, House-Brackmann facial nerve grading scale (I-VI); FU, Follow-up*.

### Study Quality

The quality of the included studies was variable (Tables 1–4). In general, the prospective comparative studies evaluating DTI and tractography in patients with trigeminal neuralgia were of high methodological quality. The most common type of comparative study encountered here were case-control studies in which the non-affected side provided the control group to which the affected side was compared. Very few studies involving patients with posterior fossa tumors included consecutive patients or unbiased assessments of study endpoints but there were notable high-quality papers involving patients with posterior fossa tumors and these have been given higher weighting in our discussion (Taoka et al., 2006; Gerganov et al., 2011; Zhang et al., 2013, 2017; Yoshino et al., 2015b; Li et al., 2017; Zolal et al., 2017a). All four cohort studies compared different methods of acquiring tractography data in patients with posterior fossa tumors. One of the listed case reports illustrating the use of tractography in a patient with a petroclival meningioma was contained within a larger technical paper comparing DTI with a more advanced multi-fiber model (HDFT: high definition fiber tractography; Yoshino et al., 2016). However, this advanced method was only used in one illustrative case that satisfied this study's inclusion criteria so the article was listed as a case report and assessed using the MINORS scoring for non-comparative studies. None of the studies included in this review documented a prospective calculation of study size.

**Table 4:** Quality of studies using MINORS criteria.

**References**	**Clear aim**	**Consecutive patients**	**Prospective data**	**Appropriate end points**	**Unbiased assessment of study endpoints**	**Appropriate follow up period**	**Loss to follow up <5%**	**Prospective calculation of study size**	**Adequate control group**	**Contemporary groups**	**Baseline equivalence of groups**	**Adequate statistical analysis**	**TOTAL**
**TRIGEMINAL NEURALGIA**													
Herweh et al., 2007	2	0	0	2	0	2	1	0	2	2	2	1	14/24
Fujiwara et al., 2011	2	2	2	2	2	2	2	0	2	2	2	2	22/24
Leal et al., 2011	2	2	2	2	2	2	2	0	2	2	2	2	22/24
Lutz et al., 2011	2	2	2	2	2	2	2	0	2	2	2	2	22/24
Hodaie et al., 2012	2	1	1	2	0	1	2	0	2	2	2	1	16/24
Liu et al., 2013	2	0	0	2	2	2	2	0	2	2	2	2	18/24
Wilcox et al., 2013	2	0	0	2	0	2	2	0	2	2	2	2	16/24
DeSouza et al., 2014	2	0	1	2	0	2	2	0	2	2	2	2	17/24
DeSouza et al., 2015	2	0	1	2	0	2	2	0	2	2	2	2	17/24
Lummel et al., 2015	2	2	2	2	2	2	2	0	2	2	2	2	22/24
Chen, D. Q. et al., 2016	2	0	0	2	0	2	2	0	2	2	2	2	16/24
Chen, S. T. et al., 2016	2	0	2	2	2	1	2	0	2	2	2	2	19/24
Lin et al., 2016	2	0	0	2	0	1	2	0	2	2	2	2	15/24
Lutz et al., 2016	2	0	2	2	2	2	2	0	2	2	2	2	20/24
Neetu et al., 2016	2	0	1	2	0	2	2	0	2	2	2	2	17/24
Hung et al., 2017	2	0	1	2	2	2	2	0	2	2	2	2	19/24
**OTHER**													
Rousseau et al., 2015	2	2	2	2	2	2	2	0	2	2	2	2	22/24
Vos et al., 2015	2	0	0	2	2	2	2	0	2	2	2	2	20/24
Coskun et al., 2017	2	0	0	0	0	1	2	0	N/A	N/A	N/A	N/A	5/16
**POSTERIOR FOSSA TUMORS**													
Taoka et al., 2006	2	0	2	2	1	2	2	0	N/A	N/A	N/A	N/A	11/16
Chen et al., 2011	2	0	2	0	0	0	0	0	N/A	N/A	N/A	N/A	4/16
Gerganov et al., 2011	2	2	2	2	2	2	2	0	N/A	N/A	N/A	N/A	14/16
Roundy et al., 2012	2	0	2	2	1	2	2	0	2	2	2	0	17/24
Zhang et al., 2013	2	2	1	2	1	2	2	0	N/A	N/A	N/A	N/A	12/16
Choi et al., 2014	2	0	2	2	0	2	2	0	N/A	N/A	N/A	N/A	10/16
Wei et al., 2015	2	2	2	2	0	2	2	0	N/A	N/A	N/A	N/A	12/16
Yoshino et al., 2015a	2	0	0	2	1	0	2	0	N/A	N/A	N/A	N/A	7/16
Yoshino et al., 2015b	2	0	0	2	2	0	2	0	2	2	2	2	16/24
Borkar et al., 2016	2	0	0	2	2	0	2	0	N/A	N/A	N/A	N/A	8/16
Hilly et al., 2016	2	0	0	2	1	0	2	0	N/A	N/A	N/A	N/A	7/16
Wei et al., 2016	2	0	0	2	0	1	2	0	N/A	N/A	N/A	N/A	7/16
Yoshino et al., 2016	2	0	2	2	0	2	2	0	N/A	N/A	N/A	N/A	10/16
Ma et al., 2016	2	0	2	2	1	2	2	0	N/A	N/A	N/A	N/A	11/16
Song et al., 2016	2	0	0	2	2	2	2	0	N/A	N/A	N/A	N/A	10/16
d'Almeida et al., 2017	2	0	0	0	0	0	2	0	N/A	N/A	N/A	N/A	4/16
Ulrich et al., 2014	2	0	1	0	0	1	2	0	N/A	N/A	N/A	N/A	6/16
Zhang et al., 2017	2	2	2	2	2	2	2	0	N/A	N/A	N/A	N/A	14/16
Zolal et al., 2017a	2	0	0	2	0	2	2	0	2	2	2	2	16/24
Zolal et al., 2017b	2	0	2	2	1	2	2	0	N/A	N/A	N/A	N/A	11/16
Li et al., 2017	2	2	2	2	2	2	2	0	N/A	N/A	N/A	N/A	14/16
Behan et al., 2017	2	0	0	2	0	2	2	0	2	2	2	0	14/24

### Image Acquisition and Processing

A variety of different MR scanners manufactured by Siemens, GE and Philips were used in the acquisition of the diffusion data. Thirty-seven studies (90%) were performed using 3T machines with the remaining 4 studies using 1.5T scanners. Most acquisition protocols used single-shot echo planar imaging (88%) with a single b-value of 1,000 s/mm^2^ (78%) but the number of gradient directions, in-plane resolution, and slice thickness varied considerably (Tables 1–3). The acquisition time was documented in a minority of studies (39%); scan time varied greatly but was typically longer in studies that used multi-shot imaging acquisition (Chen, S. T. et al., 2016; Lutz et al., 2016). Various software packages were employed for the post-processing and tractography, including MatLab (The MathWorks, Inc., Natick, MA), dTV (http://www.medimg.info.hiroshima-cu.ac.jp/dTV.II.15g/about.htm) (Masutani et al., 2003), 3D Slicer (https://www.slicer.org) (Norton et al., 2017), DTI- and DSI-Studio (http://dsi-studio.labsolver.org) (Yeh et al., 2013), MedINRIA (https://med.inria.fr) (Toussaint et al., 2007), SPM8 (https://www.fil.ion.ucl.ac.uk/spm) (Friston, 2007), iPLAN (Brainlab iPlan, Heimstetten, Germany), trackvis (http://www.trackvis.org) (Wang et al., 2007), StealthViz (Medtronic Planning Station S7, Louisville, US), DynaSuite Neuro (*in vivo* Corp.; Gainesville, USA), FSL (http://www.fmrib.ox.ac.uk/fsl) (Jenkinson et al., 2012), TEEM toolkit (https://github.com/sinkpoint/hodaie-teem) (Qazi et al., 2009), ExploreDTI (www.exploredti.com) (Leemans et al., 2009) and MRtrix3 (http://www.mrtrix.org) (Tournier et al., 2012), in addition to the hardware manufacturer's own software suites including Leonardo syngo (Siemens), FuncTool (GE), and FiberTrak (Philips) software. All studies selected regions of interest manually and most did so by using fused anatomical and diffusion images (ROIs selected using fused anatomical/diffusion imaging: 51%, diffusion imaging alone: 17%, method not specified: 32%).

### Analysis of Diffusivity Measurements

Analysis of diffusivity measurements focused on regions of the trigeminal nerve in all but one study. In the remaining study, diffusion metrics of the cochlea nerve were studied in patients with unilateral deafness, and compared to the patient's unaffected side and values in the nerves of healthy control subjects (Vos et al., 2015).

### Analysis of the Trigeminal Nerve in Patients With Trigeminal Neuralgia (TN)

Sixteen non-randomized comparative studies evaluated the diffusion metrics of the trigeminal nerve in patients with trigeminal neuralgia (TN) (Table 1). Twelve studies included healthy volunteers as a control group (Herweh et al., 2007; Fujiwara et al., 2011; Leal et al., 2011; Liu et al., 2013; Wilcox et al., 2013; DeSouza et al., 2014, 2015; Lummel et al., 2015; Chen, D. Q. et al., 2016; Lin et al., 2016; Neetu et al., 2016; Hung et al., 2017) and 4 studies compared the patient's own affected and unaffected sides (Lutz et al., 2011, 2016; Hodaie et al., 2012; Chen, S. T. et al., 2016). Two studies also included analysis of trigeminal neuralgia in patients with Multiple Sclerosis (MS) (Lummel et al., 2015; Chen, D. Q. et al., 2016), two studies evaluated patients with non-neurovascular compression TN (n-NVC-TN) (Lin et al., 2016; Neetu et al., 2016) and one study included patients with painful trigeminal neuropathy and painful temporomandibular disorders (TMD) (Wilcox et al., 2013). Analysis of diffusivity measurements was possible in all cases. Ten studies compared values in the root entry zone (REZ) (Herweh et al., 2007; Leal et al., 2011; Lutz et al., 2011, 2016; Liu et al., 2013; Wilcox et al., 2013; DeSouza et al., 2014; Lummel et al., 2015; Lin et al., 2016; Neetu et al., 2016), two studies focused on changes within the cisternal segment (Fujiwara et al., 2011; Chen, S. T. et al., 2016) and one study evaluated changes at both locations (Chen, D. Q. et al., 2016).

#### Fractional Anisotropy in TN

In the 10 studies that measured the FA in the REZ of patients with TN caused by neurovascular compression, 8 (80%) found significantly lower FA values on the affected side compared to the unaffected side (Leal et al., 2011; Lutz et al., 2011, 2016; Liu et al., 2013; DeSouza et al., 2014; Lummel et al., 2015; Chen, D. Q. et al., 2016; Neetu et al., 2016). The other two studies failed to demonstrate a statistical difference between sides (Herweh et al., 2007; Wilcox et al., 2013) but there was a trend toward lower FA values in TN-affected nerves (Herweh et al., 2007) in one of the studies. Only two studies examined diffusivity values in the cisternal segment of affected and unaffected nerves; one found no difference in FA values (Fujiwara et al., 2011) whereas the other found significantly higher FA values in the cisternal segment of affected nerves (Chen, D. Q. et al., 2016). Chen et al were also the only group to compare diffusivity values in the REZ and cisternal segment and found that TN-affected nerves appeared to have higher FA in the cisternal segment and lower FA in the REZ when compared to the patient's unaffected side (Chen, D. Q. et al., 2016).

#### Apparent Diffusion Coefficient and Mean Diffusivity in TN

Seven studies (44%) examined changes in ADC values within TN-affected nerves; four (57%) found ADC to be significantly higher in the REZ of affected nerves (Leal et al., 2011; Lummel et al., 2015; Chen, S. T. et al., 2016; Neetu et al., 2016) whereas the remaining three studies found no difference (Fujiwara et al., 2011; Lutz et al., 2011, 2016). Five studies (31%) examined changes in MD; four found significantly higher MD values in the REZ of idiopathic TN-affected nerves (Liu et al., 2013; DeSouza et al., 2014, 2015; Chen, D. Q. et al., 2016) and one found no statistical difference (Wilcox et al., 2013); however, two of the studies only demonstrated a statistically higher MD value when the REZ of affected nerves was compared to healthy controls with no statistical difference noted when compared to the unaffected side of patients with TN (DeSouza et al., 2014, 2015).

#### Radial and Axial Diffusivity in TN

Six studies (75%) reported RD and AD values in the affected and unaffected nerves of patients with idiopathic TN (Liu et al., 2013; DeSouza et al., 2014, 2015; Chen, D. Q. et al., 2016; Chen, S. T. et al., 2016; Lin et al., 2016) and a further two studies studied changes in these diffusivity metrics in patients following treatment (Hodaie et al., 2012; Hung et al., 2017). All five studies that examined changes in the REZ of TN-affected nerves reported significantly higher RD values in the REZ compared to the nerves of healthy controls (Liu et al., 2013; DeSouza et al., 2014, 2015; Chen, D. Q. et al., 2016; Lin et al., 2016) but significantly lower RD values were observed in the cisternal segments of affected nerves (Chen, D. Q. et al., 2016; Chen, S. T. et al., 2016). No significant differences were observed in the RD values of TN-affected nerves when compared to the unaffected side in two studies (DeSouza et al., 2014, 2015). Within the REZ it is likely that a change in RD is the main driver of a reduced FA which most likely represents a decrease in axonal integrity rather than a reduced alignment or coherence however further work is required to establish the nature of changes seen within the cisternal segment.

Fewer studies observed a statistically significant difference in AD values: three studies observed higher AD values in the REZ of TN-affected nerves when compared to the nerves of healthy controls and two studies observed no difference in AD values (Liu et al., 2013; Lin et al., 2016). There were no significant changes reported in AD when the REZ of affected nerves were compared to the unaffected side in patients with idiopathic TN but two studies demonstrated significantly higher AD values when TN-affected nerves were compared with the REZ of the nerves of healthy controls (DeSouza et al., 2014, 2015). One of these studies also analyzed changes in the cisternal segment of the nerve and demonstrated significantly lower AD values in the TN-affected nerves when compared to the unaffected side (Chen, D. Q. et al., 2016).

#### Diffusivity Changes in Patients With Multiple Sclerosis and TN

In patients with MS-associated TN, Lummel et al. found that FA was significantly lower and ADC higher in the REZ of both the TN-affected and unaffected sides when compared to unaffected side of patients with idiopathic TN or healthy controls (Lummel et al., 2015). Chen et al examined different diffusivity values in various nerve segments of patients with idiopathic and MS-associated TN (Chen, D. Q. et al., 2016). They demonstrated FA values were significantly lower in the peri-lesional segments of TN-affected nerves compared to the unaffected side of MS-associated TN patients and significantly lower than the nerves of patients with idiopathic TN and healthy controls. No significant differences were noted in other diffusivity values (MD, RD, or AD) within the peri-lesional segments of MS-associated TN nerves compared to the nerves of patients with idiopathic TN or healthy controls but AD and RD were shown to be significantly higher in the REZ of patients with both idiopathic and MS-associated TN (Chen, D. Q. et al., 2016).

#### Diffusivity Changes in Patients With TN Following Treatment

Five studies examined differences in the diffusion characteristics of patients' affected trigeminal nerves before and after treatment including microvascular decompression (MVD) (Fujiwara et al., 2011; DeSouza et al., 2015; Hung et al., 2017), Gamma Knife stereotactic radiosurgery (GK SRS) (Hodaie et al., 2012; DeSouza et al., 2015; Hung et al., 2017) and radiofrequency ablation (RFA) (Chen, S. T. et al., 2016). All four studies with diffusivity data from the REZ demonstrated lower FA values in the nerves of those patients who responded to treatment (Fujiwara et al., 2011; Hodaie et al., 2012; DeSouza et al., 2015; Hung et al., 2017) with a trend toward FA reduction demonstrated in the cisternal segment following effective treatment (Chen, S. T. et al., 2016). Effective treatment also appears to reduce other diffusivity changes seen in TN-affected nerves (Hodaie et al., 2012; DeSouza et al., 2015). Most recently, Hung et al demonstrated that treatment outcomes may be predicted by alterations in pre-surgical diffusivity measurements with long-term treatment responders having lower cisternal AD and MD values and non-responders having lower FA values in the REZ and higher AD values in the pontine segment (Hung et al., 2017).

### Analysis of the Trigeminal Nerve in Other Pathologies

Rousseau et al. examined the potential impact of recurrent HSV and VSV keratitis on the axonal architecture of trigeminal nerves by assessing changes in the diffusivity metrics of patients' trigeminal nerves (Rousseau et al., 2015). They discovered FA to be significantly lower in the REZ of trigeminal nerves on the ipsilateral side to the affected event and demonstrated that the asymmetry was more than the intra-individual variability in controls (Rousseau et al., 2015). No such significant differences were observed in ADC values.

Coskun et al. reported diffusivity analysis in 2 patients with SUNCT, again demonstrating FA to be lower on the affected side (Coskun et al., 2017). ADC values were higher in the affected side in one patient with no difference observed in the other (Coskun et al., 2017).

### Analysis of the Cochlear Nerve

Vos et al. assessed diffusivity changes in the cochlear nerves of patients with profound unilateral sensorineural hearing loss (Vos et al., 2015). Here, the authors assumed any changes in DTI metrics of the vestibulocochlear nerve would reflect changes in the cochlear nerve as it is the largest nerve and no changes are expected in the facial or vestibular nerves in unilateral deafness. They reported no significant difference in diffusivity values between patients' deaf-sided and healthy-sided cochlear nerves but there was a small but significant reduction in FA values in both cochlear nerves in patients compared with normal-hearing controls (Vos et al., 2015).

### Fiber Tractography

**Figure 3:**
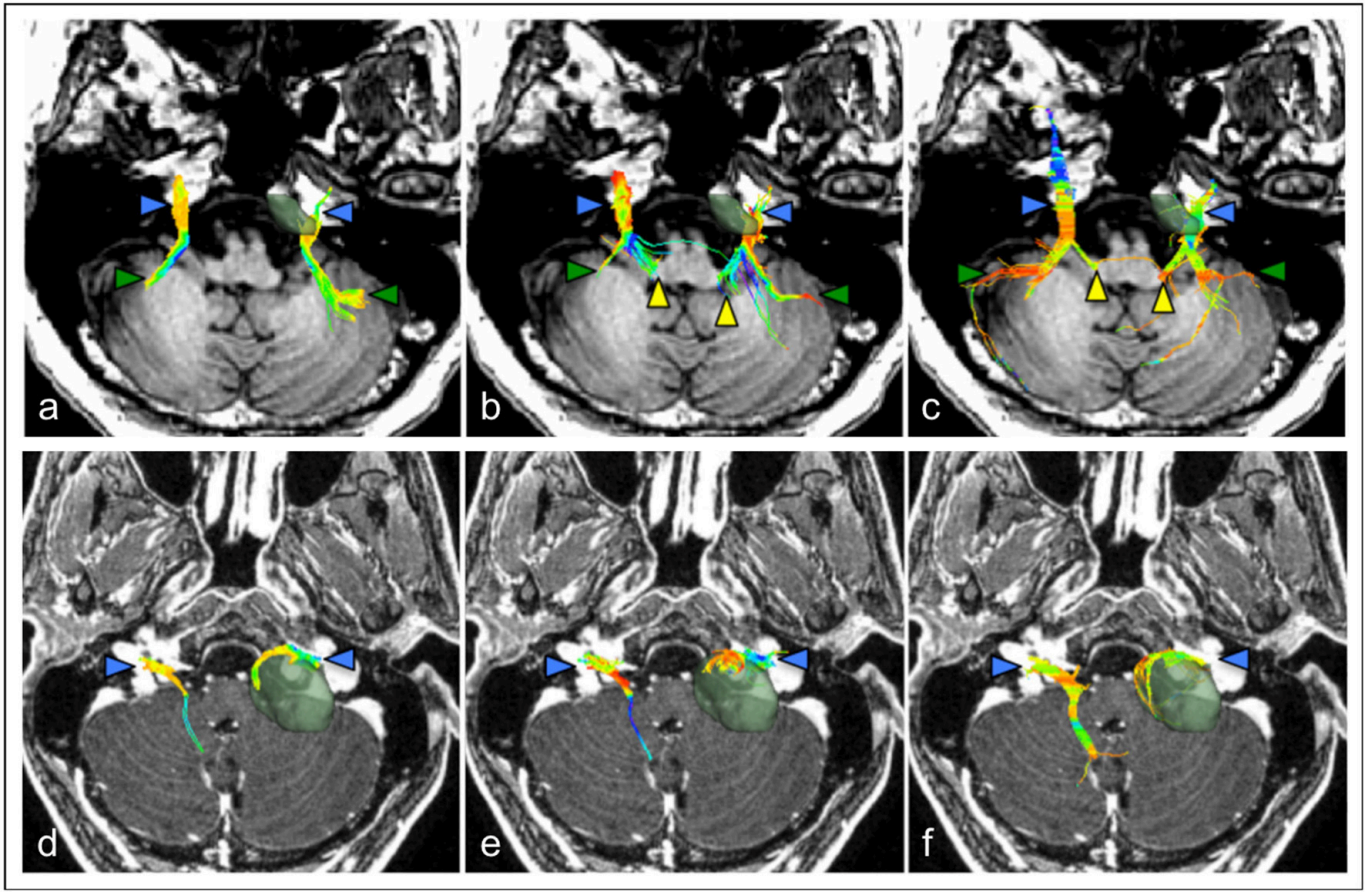
Comparison of cranial nerve fiber tractography methods in patients with posterior fossa tumors. Streamlines are displayed overlaid on a T1 anatomical image. Color triangles indicate particular anatomical landmarks: blue: cranial nerves, green: superior cerebellar fibers, yellow: brainstem nuclei. **(a–c)** Tractography of the trigeminal nerve (CN V) in a patient with a left-sided petroclival meningioma; **(d–f)** Tractography of the facial-vestibulocochlear bundle (CN VII/VIII) in a patient with a left sided vestibular schwannoma. **(a,d)** single diffusion tractography, **(b,e)** extended streamline tractography, **(c,f)** constrained spherical deconvolution. *Images courtesy of* (Behan et al., 2017) *(CC-BY license)*.

Fiber tractography of one or more cranial nerves was performed in 30 studies (73%). All 22 studies involving patients with brain tumors focused on generating tractography of the surrounding cranial nerves. Eighteen of those (82%) involved patients with a vestibular schwannoma (Figure 3) but cranial nerve tractography was also assessed in patients with trigeminal schwannomas (Wei et al., 2016), meningiomas (Ma et al., 2016; Yoshino et al., 2016; Behan et al., 2017; d'Almeida et al., 2017; Zolal et al., 2017a), brainstem cavernomas (Ulrich et al., 2014) and other unspecified cerebellopontine angle tumors (Roundy et al., 2012). Given the functional importance of preserving the facial nerve during surgery, almost all tumor studies (21/22, 95%) included tractography of CN VII or the VII/VIII complex, but tractography of other cranial nerves within the posterior fossa has also been demonstrated including CN IV (Ma et al., 2016; Yoshino et al., 2016), CN V (Chen et al., 2011; Ulrich et al., 2014; Wei et al., 2016; Yoshino et al., 2016; Behan et al., 2017; Zolal et al., 2017a) and CN VI (Ulrich et al., 2014; Yoshino et al., 2016). Eight non-tumor studies reported tractography results; seven studies detailed tractography of the trigeminal nerve (Fujiwara et al., 2011; Hodaie et al., 2012; DeSouza et al., 2014; Rousseau et al., 2015; Chen, D. Q. et al., 2016; Coskun et al., 2017; Hung et al., 2017) [five of which were in patients with TN (Fujiwara et al., 2011; DeSouza et al., 2014; Chen, D. Q. et al., 2016; Hung et al., 2017)] with the other study focusing on the cochlea nerve (Vos et al., 2015).

A deterministic method of generating fiber tractography was used in the majority of studies (93%). The reported success rate in generating fiber tracts of the cranial nerves within the posterior fossa varied considerably (0–100% success) but was not lower in those studies imaging smaller nerves [100% success rate was reported in all studies imaging trochlear (Ma et al., 2016; Yoshino et al., 2016) or abducens nerve (Ulrich et al., 2014; Yoshino et al., 2016)]; however, further studies are needed to confirm this observation. Zolal et al. recently published their experience using probabilistic methods (Zolal et al., 2017a,b). One of their studies provided a comparison of both techniques and appeared to suggest that probabilistic tracking was more effective at depicting the cranial nerves (Zolal et al., 2017a). In 15 studies (50%), a single region of interest (ROI) was used to seed the tractography, whereas 12 studies (40%) used a two ROIs, one to seed and one to select [methods not specified in 3 studies]. Details of thresholding was available in 25 of the 30 studies that performed tractography. A variety of fixed FA thresholds (range 0.02–0.20) were chosen in 16/25 studies (64%) (Taoka et al., 2006; Chen et al., 2011; Fujiwara et al., 2011; Gerganov et al., 2011; Hodaie et al., 2012; Roundy et al., 2012; Zhang et al., 2013; DeSouza et al., 2014; Rousseau et al., 2015; Borkar et al., 2016; Chen, D. Q. et al., 2016; Hilly et al., 2016; Ma et al., 2016; Song et al., 2016; Behan et al., 2017; Hung et al., 2017) with a variable approach to selecting the FA threshold used in the other 9 studies (36%) (Choi et al., 2014; Wei et al., 2015; Yoshino et al., 2015a,b, 2016; Li et al., 2017; Zhang et al., 2017; Zolal et al., 2017a,b), including the two studies that employed diffusion spectrum imaging (DSI).

In studies that involved patients undergoing surgery, fiber tractography of the facial nerve was correlated with the surgeon's intraoperative finding in 19 studies (86%) but the reported accuracy of facial nerve tractography was extremely variable (17–100%) (Table 3). The accuracy of the tractography was assessed by the operating surgeon inspecting and documenting the location of the nerve in relation to the tumor and was usually assisted by the use of qualitative electrophysiological monitoring (79% of studies). Recently, Li et al also managed to quantitatively correlate electrophysiological results by registering the points of stimulation with the patient's tractography results using an intraoperative neuronavigation system (Li et al., 2017). Several studies reported patients' post-operative facial nerve status but none of them evaluated the effectiveness of using fiber tractography to prevent facial nerve injury.

## Discussion

There is a clinical need to improve the visualization of cranial nerves within the posterior fossa, particularly in the context of pathology, and to enable detailed analysis of the affected nerves' microstructure. In normal anatomy, the visualization of cranial nerves from the brainstem to the skull base is currently optimized on high contrast T2 sequences (Figure 1) but it is still extremely difficult to image the cranial nerves as they traverse the skull, and in patients with associated compressive tumor pathology our experience is that anatomical T2 imaging cannot depict the course of adjacent nerves. The prospect of obtaining 7T MR imaging in the routine clinical setting should improve the ability to reliably visualize the larger cranial nerves such as the oculomotor (CN III), trigeminal (CN V) and vestibulocochlear (CN VIII) through the brain's cisterns but anatomical constraints are still likely to impede the visualization of smaller nerves beyond the skull base and in the context of associated pathology such as compressive brain tumors. Consequently, the current focus of this review is to examine the effectiveness of utilizing diffusion MRI to image the cisternal segments of the cranial nerves within the posterior fossa.

Forty-one studies met the study's inclusion criteria and were qualitatively analyzed. The methodological quality of the included studies varied considerably but the available evidence demonstrates that it is possible to acquire diffusion MRI data using a variety of clinical scanners and process this data with various software packages, none of which were shown to be more, or less, effective in this respect. Most clinical studies examining the use of diffusion MRI in patients with cranial nerve pathologies have either focused on performing DTI analyses of the trigeminal nerve in patients with trigeminal neuralgia or generating DTI-based tractography of the cranial nerves in patients with posterior fossa tumors.

### Trigeminal Neuralgia

Sixteen studies evaluated diffusivity changes in the trigeminal nerve of patients suffering from trigeminal neuralgia. The trigeminal nerve is the largest cranial nerve within the posterior fossa and all studies successfully managed to acquire diffusion MRI data of adequate quality for tractography and/or quantitative DTI analysis. Ten studies examined changes in FA within the REZ of the trigeminal nerve compared to the patient's unaffected side; lower FA values demonstrated within the REZ of TN-affected nerves in all ten studies and a statistically significant decrease observed in 80% (Table 1). However, due to the heterogeneous way data was presented in the various studies, it was not possible to quantitatively calculate the expected difference in FA values between TN-affected and unaffected nerves. A variety of other diffusivity metrics were also analyzed by different studies but no conclusive statements may be drawn from the current evidence given the small number of studies involved and the varying formats in which results were presented. It appears that RD values within the REZ of TN-affected are significantly higher than the unaffected nerves of healthy control subjects (Liu et al., 2013; DeSouza et al., 2014, 2015; Chen, D. Q. et al., 2016; Chen, S. T. et al., 2016; Lin et al., 2016) but De Souza et al. found no significant difference when comparing values with the patient's unaffected side (DeSouza et al., 2014, 2015). Likewise, mixed results were reported in the small number of studies that examined differences in AD values in patients with trigeminal neuralgia.

Interesting observations were noted in the studies that compared diffusivity values in different segments of the nerve (Fujiwara et al., 2011; Chen, D. Q. et al., 2016; Hung et al., 2017) and in the two studies that compared the diffusion metrics of affected trigeminal nerves in patients with MS-associated TN and idiopathic trigeminal neuralgia (Lummel et al., 2015; Chen, D. Q. et al., 2016). In particular, further work is needed to establish whether TN-affected nerves do indeed have a higher FA in the cisternal segment and lower FA in the REZ compared to unaffected nerves (Chen, D. Q. et al., 2016) and whether alterations in pre-surgical diffusivity measurements may be used as a predictive tool to prognosticate surgical response (Hung et al., 2017). In light of Hung et al.'s recent findings that treatment outcomes may be predicted by alterations in pre-surgical diffusivity measurements it would also be worthwhile evaluating if patients with different diffusion signatures respond differently to different treatment modalities. If so, diffusion metrics may also be used to guide treatment choice as well as prognosticating an individual patient's response.

### Cranial Nerve Tractography in Patients With Posterior Fossa Tumors

Taoka et al. (2006) were the first to demonstrate the feasibility of using preoperatively-acquired DTI fiber tractography to delineate the course of the VII-VIII cranial nerve complex around a vestibular schwannoma. Since their initial work a further 21 studies have assessed the effectiveness of generating cranial nerve tractography in patients with posterior fossa tumors, the majority of whom had a vestibular schwannoma (82%). Nearly all studies used a deterministic tractography approach. Varying numbers of ROIs were used to select the fiber tracts and different methods of selecting an FA threshold were employed (Table 3). Overall, the success rate in generating tractography was extremely variable (0–100% success rate) and correlation with the surgeon's finding was also inconsistent (17–100% accuracy across all studies). However, it is important to note that failure to generate any fiber tracts was only demonstrated in one study using standard DTI acquisition and when Roundy et al. performed higher resolution diffusion imaging, tractography was successfully generated in all patients (Roundy et al., 2012). Similarly, a 17% accuracy rate was reported by Yoshino et al. when standard DTI was used but this increased to 67% when DTI was combined with multifused CE-FIESTA images (Yoshino et al., 2015a). Excluding these results, the success rate of generating cranial nerve tractography in patients with a posterior fossa tumor rises to 82–100%, similar to that reported by Ung et al. in their brief review of the literature (Ung et al., 2016); however, intraoperative accuracy remains inconsistent (30–100%).

In recent years, several groups have attempted to improve the accuracy of cranial nerve tractography by various means. Wei et al. described a method of “superselective” tracking whereby optimal maps only containing bundles of axons with the lowest density, originating from the brainstem were selected in order to better delineate the anatomical relationship between the bundle and surrounding tissues (Wei et al., 2015). Various FA values were also used to identify the maximal FA value of each fiber. A similar method was replicated in two recent studies with good results (Li et al., 2017; Zhang et al., 2017) although this technique is very time consuming and still does not allow one to specifically distinguish the facial nerve from within the facial-vestibular (CN VII-VIII) complex. Furthermore, manual fiber selection and *ad-hoc* threshold manipulations are mainly based on investigator expectation of the anatomical position of the nerve, and as Zolal et al. highlighted in their recent article (Zolal et al., 2017a), such a method weakens the claims about reliable cranial nerve detection.

Two studies used diffusion spectrum imaging to generate fiber tractography of the cranial nerves (Yoshino et al., 2016; Zolal et al., 2017a), which is one of a number of more complex diffusion techniques that has been developed to address some of the limitations of the diffusion tensor model. DSI requires many more gradient directions along multiple *b*-values and a higher maximum *b*-value to generate the desired orientation information and as such is challenging to do in a clinical setting. Yoshino et al suggested that DSI may have the potential to distinguish the facial nerve from the vestibulocochlear nerve and accurately detect cranial nerve position (Yoshino et al., 2016) although this was not demonstrated in the current study. Zolal et al. subsequently compared the depiction of cranial nerves II, III, V, and the VII+VIII complex using both deterministic and probabilistic methods in a cohort of 30 healthy subjects obtained from the Kirby Repository (KR) and HCP databases (Zolal et al., 2017a). In both instances, for tracking the VII-VIII complex, ROIs were set at the brainstem and in the internal auditory meatus. This study confirmed that using diffusion MRI data with higher angular resolution and overall better quality led to better depictions of the nerves, confirming the finding previously reported by Roundy et al. (2012). Yoshino et al first described this method of gradually increasing the FA threshold in 2015 in deterministic fiber tracking (Yoshino et al., 2015a) and similar results were obtained in the studies utilizing DSI. Probabilistic index of connectivity (PICo maps) were created for each of the nerves, and to find the optimal probability threshold, the PICo maps were filtered at threshold values of 0.05–0.95 in steps of 0.05. Zolal et al. concluded that probabilistic tracking with a gradual PICo threshold increase is more effective at depicting the cranial nerves than the previously described deterministic tracking because it eliminates the erroneous fibers without manual intervention. A small limitation of this method is the increased computational time required in using the probabilistic method (30 min per nerve vs. 15 min per nerve for deterministic tracking) but in our opinion, such a difference is unlikely to impact clinical use given that image processing and fiber tractography is rarely required in real-time.

Recently, Behan et al compared three distinct reconstruction methods to generate tractography of the cranial nerves in patients with associated posterior fossa tumors, including conventional diffusion tensor tractography, a two-tensor reconstruction method (eXtended streamline tractography, XST), and a fiber orientation distribution-based method (constrained spherical deconvolution, CSD) (Figure 3) (Behan et al., 2017). They found that XST and CSD-based reconstruction methods produced more detailed projections of CN V and CN VII/VII compared to DTI tractography but CSD-based methods appeared to generate more invalid streamlines. Consequently, the authors favored using XST to visualize the cranial nerves in patients with posterior fossa tumors however a reliable and accurate method to separately depict the facial nerve in these patients is still required.

In a separate article, Zolal et al. used probabilistic tracking to generate preoperative tractography of the facial-cochlear complex in 21 patients undergoing vestibular schwannomas surgery with the accuracy determined intraoperatively by surgical inspection and the use of qualitative electrophysiological monitoring (Zolal et al., 2017a). Preoperative tractography was accurate in 81% of cases but many of the results also contained false-positive pathways, typically dorsal to the tumor. Probabilistic tractography is highly reliant on proper thresholding to achieve high-quality reconstruction of biological pathways and, consequently, tend to generate more invalid fiber bundles compared to deterministic tractography methods (Maier-Hein et al., 2017). In their study, Zolal et al speculated that this was because probabilistic tracking preferentially tracks large axonal bundles which in the case of patients with a vestibular schwannoma includes the larger vestibulocochlear nerve.

## Limitations

The present systematic review was limited by various factors. Firstly, given the variety of ways diffusion data was presented and the small numbers of available studies, it was not possible to perform a meta-analysis and quantitatively analyse the data to draw any firm conclusions concerning diffusivity changes observed in the trigeminal nerve of patients with TN. Secondly, studies evaluating cranial nerve tractography were of mixed methodological quality and used a variety of acquisitions, limiting our discussion to a qualitative report of a small number of higher quality studies.

## Conclusion

Current work suggests that fiber tractography has the ability to delineate the course of individual nerves within the posterior fossa. Further work is required to incorporate cranial nerve tractography into the intraoperative workflow and new avenues of using diffusion MRI should be explored to optimize and improve its reliability. In particular, new techniques of delineating the facial nerve from the VII-VIII complex should be examined and validated with the use of quantitative intraoperative electrophysiological measurements. Diffusion MRI has the potential to inform our understanding of the microstructural changes that occur within the cranial nerves in various pathologies and may eventually be able to assist clinicians to deliver individualized treatment plans.

## Author Contributions

JS conceived the manuscript. JS and SV performed the systematic literature search. JS drafted the initial manuscript. SV, TV, RB, SS, SB, and SO reviewed the final manuscript.

### Conflict of Interest Statement

The authors declare that the research was conducted in the absence of any commercial or financial relationships that could be construed as a potential conflict of interest.
